# Mol­ecular structure and selective theophylline com­plexation by conformational change of diethyl *N*,*N*′-(1,3-phenyl­ene)dicarbamate

**DOI:** 10.1107/S2053229624003358

**Published:** 2024-05-07

**Authors:** Juan Saulo González-González, Alfonso Martínez-Santos, María José Emparán-Legaspi, Armando Pineda-Contreras, Francisco Javier Martínez-Martínez, Marcos Flores-Alamo, Hector García-Ortega

**Affiliations:** a Instituto de Farmacobiología, Universidad de la Cañada, Carretera Teotitlán-San Antonio Nanahuatipán, km 1.7 s/n, Teotitlán de Flores Magón, Oaxaca 68540, Mexico; b Facultad de Ciencias Químicas, Universidad de Colima, km 9, Carretera Colima-Coquimatlán, Coquimatlán, Colima 28400, Mexico; c Facultad de Química, Universidad Nacional Autónoma de México, Ciudad de México 04510, Mexico; Cinvestav, Mexico

**Keywords:** mechanochemistry, crystal structure, host-guest com­plex, phenyl carbamate, conformational change, IR spectroscopy, theophylline

## Abstract

The receptor ability of diethyl *N*,*N*′-(1,3-phenyl­ene)dicarbamate (**1**) to form host–guest com­plexes with theophylline (TEO) and caffeine (CAF) by mechanochemistry was evaluated. The formation of the **1**–TEO com­plex (C_12_H_16_N_2_O_4_·C_7_H_8_N_4_O_2_) was preferred and involves the conformational change of one of the ethyl carbamate groups of **1** from the *endo* conformation to the *exo* conformation to allow the formation of inter­molecular inter­actions. The formation of an N—H⋯O=C hydrogen bond between **1** and TEO triggers the conformational change of **1**. CAF mol­ecules are unable to form an N—H⋯O=C hydrogen bond with **1**, making the conformational change and, therefore, the formation of the com­plex impossible. Conformational change and selective binding were monitored by IR spectroscopy, solid-state ^13^C nuclear magnetic resonance and single-crystal X-ray diffraction. The **1**–TEO com­plex was characterized by IR spectroscopy, solid-state ^13^C nuclear magnetic resonance, powder X-ray diffraction and single-crystal X-ray diffraction.

## Abstract 2

The diethyl *N*,*N*′-(1,3-phenyl­ene)dicarbamate–theophylline (**1**–TEO) com­plex was obtained by mechanochemistry involving the conformational change of one of the ethyl carbamate groups of **1** from the *endo* conformation to the *exo* conformation to allow the formation of inter­molecular inter­actions.

## Introduction

Host–guest com­plexes are supra­molecular species formed by two or more mol­ecules or ions stabilized by noncovalent inter­actions (principally hydrogen bonds) involving mol­ecular recognition between the functional groups of both. A host (or receptor) is a mol­ecule with a cavity suitable for guest binding. The design of mol­ecular receptors involves an understanding of the inter­molecular inter­actions using building blocks with functional groups that allow the binding of specific guests (or substrates). The study of host–guest com­plexes in solution and the solid state has allowed its application in various fields, such as drug delivery systems (Wankar *et al.*, 2020 ▸), mol­ecular diagnostics (Yu & Chen, 2019 ▸), biomaterials (Webber *et al.*, 2016 ▸), artificial mol­ecular machines (Erbas-Cakmak *et al.*, 2015 ▸), sensors (Kim *et al.*, 2012 ▸) and biosensors (Lim *et al.*, 2021 ▸).

Mol­ecules with the amide group [*R*′–NH–(C=O)–*R*] have been used in the design of mol­ecular receptors due to their ability to act as a donor and acceptor of hydrogen bonds in the formation of supra­molecular com­plexes. These amide receptors have been exploited in a cyclic and acyclic manner using functionalities such as carboxamides (Bondy & Loeb, 2003 ▸), ureas (dos Santos *et al.*, 2008 ▸), oxalamates (González-Gon­zález *et al.*, 2014 ▸), amino acids (Kubik & Mungalpara, 2017 ▸) and carbamates (Saucedo-Balderas *et al.*, 2015 ▸), which have been studied in the formation of supra­molecular com­plexes with anions, polyphenols, amino acids and pharmaceutical ingredients (Siering *et al.*, 2006 ▸).

Phenyl carbamate is an organic group used in drug design with biological applications, such as acetyl­cholinesterase inhibitors for the treatment of Alzheimer’s disease (Colović *et al.*, 2013 ▸; Krátký *et al.*, 2016 ▸), anti­parasitic agents (Angeles *et al.*, 2000 ▸; Jiménez-Cardoso *et al.*, 2004 ▸) and anti­convulsants (Matošević & Bosak, 2020 ▸). In organic synthesis they are used as precursors of iso­cyanates (Baba *et al.*, 2005 ▸; Sun *et al.*, 2013 ▸) and in the chiral separation of anti­fungal agents (Ali *et al.*, 2021 ▸).

The chemical structure of phenyl carbamates includes carbonyl (C=O) and amino (N—H) groups, which can form inter- and intramol­ecular hydrogen-bond inter­actions. Also π-inter­actions can be formed by the phenyl ring (Matošević & Bosak, 2020 ▸). Supra­molecular studies of phenyl carbamates (Shahwar *et al.*, 2009 ▸; AaminaNaaz *et al.*, 2017 ▸) are focused on the self-assembly of crystal structures, revealing that the N—H⋯O=C hydrogen-bond inter­action drives the supra­molecular architecture in the solid state, leading to the formation of supra­molecular chains in phenyl carbamate derivatives, and supra­molecular columns in phenyl­enebis-carbamates (García-Báez *et al.*, 2004 ▸; Lu *et al.*, 2005*a* ▸,*b* ▸).

Theophylline (bronchodilator) and caffeine (nervous system stimulant) are pharmacologically active mol­ecules (Boushey, 2012 ▸) that possess functional groups (C=O and N—H in only TEO) capable of forming noncovalent inter­actions which have been applied in the development of mol­ecular receptors for the mol­ecular recognition of TEO and CAF due to its potential biomedical and industrial applications (Sahoo, 2015 ▸).

The formation of supra­molecular com­plexes has allowed the identification and qu­anti­fication of com­pounds of pharmaceutical inter­est. To evaluate the ability of diethyl *N*,*N*′-(1,3-phenyl­ene)dicarbamate (**1**) as a receptor to form host–guest com­plexes, we report here the mechanochemical com­plexation of **1** with theophylline (TEO) and caffeine (CAF) (Scheme 1). The obtained **1**–TEO com­plex was prepared by solvent-assisted grinding and was characterized by IR spectroscopy (IR), powder X-ray diffraction (PXRD) and solid-state ^13^C nuclear magnetic resonance (NMR). The mol­ecular structure was obtained by single-crystal X-ray diffraction.

## Experimental

### com­pounds

1,3-Phenyl­enedi­amine, ethyl chloro­formate, tri­ethyl­amine, tetra­hydro­furan (THF) anhydrous, dimethyl sulfoxide (DMSO) anhydrous and theophylline anhydrous were purchased from Aldrich. Chloro­form, di­chloro­methane, me­tha­nol and aceto­nitrile of ACS grade were purchased from Química Mayer. Caffeine was purchased from BASF. All the reagents were used as received.

### Synthesis of diethyl *N*,*N*′-(1,3-phenyl­ene)dicarbamate, 1

A mixture of 1,3-phenyl­enedi­amine (3.0 g, 27.7 mmol) and tri­ethyl­amine (61.0 mmol, 8.5 ml) in tetra­hydro­furan (THF, 250 ml) was placed in an ice bath. After 10 min of stirring, ethyl chloro­formate (5.3 ml, 61.0 mmol) was added dropwise. The mixture was stirred for 24 h at room temperature and then filtered to obtain a THF solution which was evaporated to dryness. The obtained solid was solubilized in chloro­form and filtered to separate the insoluble solid. The chloro­form solution was evaporated to obtain a solid corresponding to com­pound **1**. 
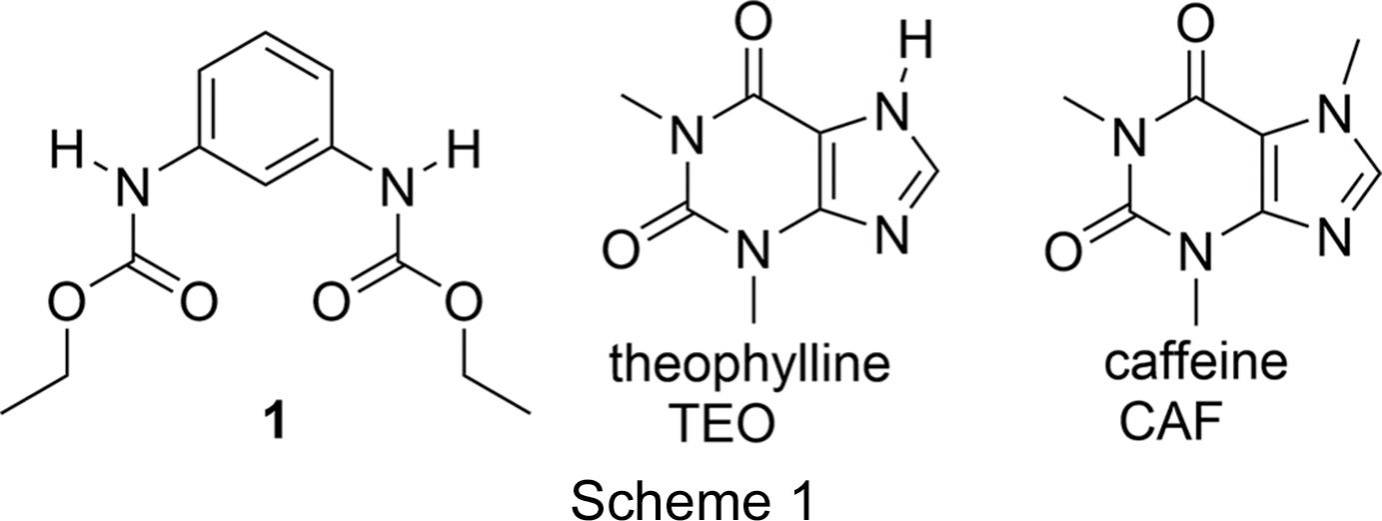


Analytical data for **1**: yield 53.17%; white solid; m.p. 146–148 °C; IR (ATR): ν (cm^−1^) 3283 (N—H), 1704, 1688 (C=O). ^1^H NMR (DMSO-*d*_6_, 400 MHz, δ ppm): 9.55 (*s*, 2H, N–H7), 7.70 (*s*, 1H, H2), 7.07 (*dd*, 2H, *J* = 7.0, 2.2 Hz, H4, H6), 7.13 (*t*, 1H, *J* = 6.9 Hz, H5). ^13^C NMR (DMSO-*d*_6_, 100 MHz, δ ppm): 153.9 (C8), 140.0 (C1, C3), 129.1 (C2), 113.1 (C4, C6), 60.4 (C10), 14.9 (C11). Analysis calculated (%) for C_12_H_16_N_2_O_4_: C 57.13, H 6.93, N 11.10; found: C 56.82, H 6.39, N 11.04.

### Mechanochemical synthesis and crystallization

A mixture in a 1:1 molar ratio of **1** (0.30 g, 1.18 mmol) and TEO (0.21 g, 1.18 mmol) was placed in a porcelain mortar. Before starting the grinding with a pestle, 0.5 ml of di­chloro­methane was added and the mixture was ground for 3 min. At the end of the grinding time, the di­chloro­methane was evaporated and the ground powder was collected in the centre of the mortar. The cycle of adding 0.5 ml of di­chloro­methane and grinding for 3 min was repeated three more times until 12 min of grinding time was com­pleted. After 12 min of grinding time, the obtained ground powder was stored in a glass vial. **1**–CAF ground powder was obtained by grinding **1** (0.30 g, 1.18 mmol) and CAF (0.22 g, 1.18 mmol) under the same conditions as described for the **1**–TEO mixture.

Solutions of **1** and **1**–TEO were prepared by dissolving the powder of **1** in DMSO and the ground powder of **1**–TEO in a 1:1 methanol/aceto­nitrile mixture. Single crystals were ob­tained after evaporation of the solvent.

### Instrumentation

The IR spectra of solids **1**, TEO, **1**–TEO ground powder, **1**–TEO single crystal, CAF and **1**–CAF ground powder were obtained in a Bruker Tensor-27 spectrophotometer equipped with an attenuated total reflectance (ATR) system (16 scans, spectral range 600–4000 cm^−1^, resolution 4 cm^−1^).

Powder X-ray diffraction patterns of solids **1**, TEO, polycrystalline **1**–TEO ground powder, CAF and **1**–CAF polycrystalline ground powder were collected on a PANalytical X′pert Pro diffractometer with Cu *K*α_1_ radiation (λ = 1.5405 Å, 45 kV, 40 mA) from 5.0 to 50.0° in 2θ.

Solution ^1^H and ^13^C NMR spectra of **1** were recorded on a Bruker 400 Avance III spectrometer (^1^H = 400 MHz and ^13^C = 100 MHz) at room temperature (25 °C) using DMSO-*d*_6_ as solvent and SiMe_4_ as the inter­nal reference (the NMR spectra of **1** are shown in Figs. S1 and S2 in the supporting information). Solid-state cross-polarization/magic angle spinning (CP/MAS) ^13^C spectra of **1**, TEO and the polycrystalline ground powder of **1**–TEO were recorded on a Bruker 400 Avance III (^13^C = 100 MHz) instrument at 25 °C, using 4 mm bullet-type Kel-F zirconia rotors with a spinning rate of 8 kHz and an acquisition time of 32 ms. The recycle time of the pulse was 3 s. An adamantane signal was used as the external reference (δ = 38.48 ppm). Processing of the NMR spectra was performed with *MestReNova* software (Version 14.2.0-26256; Mestrelab Research, 2021 ▸).

Elemental analysis of **1** was performed using a vario MICRO Cube CHN(S) analyzer (Fig. S3 in the supporting information).

The melting point (m.p.) of **1** was measured using an Electrothermal IA9300 apparatus and is uncorrected.

### Refinement

Crystal data, data collection and structure refinement details are summarized in Table 1 ▸. The H atoms of the amine group (H—N) were located in a difference map and refined isotropically with *U*_iso_(H) = 1.2*U*_eq_(N) for H—N hydrogens. H atoms attached to C atoms were placed in geometrically idealized positions and refined as riding on their parent atoms, with C—H = 0.93–0.99 Å and *U*_iso_(H) = 1.2*U*_eq_(C) for aromatic and methyl­ene groups, and 1.5*U*_eq_(C) for methyl groups.

## Results and discussion

### IR spectroscopy

**Figure 1:**
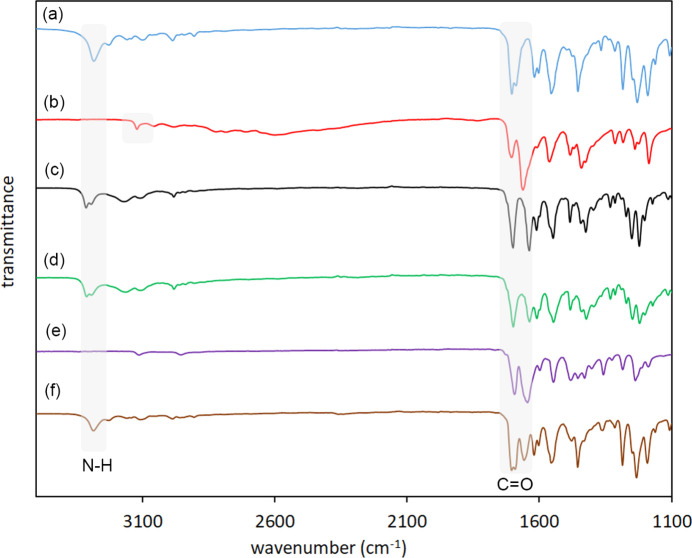
The IR spectra of (*a*) **1**, (*b*) TEO, (*c*) the polycrystalline powder of **1**–TEO after 12 min of grinding, (*d*) a single crystal of **1**–TEO, (*e*) CAF and (*f*) the ground powder of **1**–CAF.

The IR spectra of **1**, TEO and CAF (González-González *et al.*, 2017 ▸) were com­pared with the IR spectra of the polycrystalline ground powders (**1**–TEO and **1**–CAF) and the single crystal of **1**–TEO (the IR frequencies are listed in Table 2 ▸). The formation of the **1**–TEO powder com­plex was evidenced by the shift of the N—H and C=O stretching bands in the IR spectrum of the **1**–TEO ground powder with respect to the starting com­pounds, suggesting the formation of inter­molecular N—H⋯O=C hydrogen bonds (Fig. 1 ▸). On the other hand, the IR spectrum of the **1**–CAF ground powder did not show shifts with respect to the starting materials, suggesting that the formation of the **1**–CAF com­plex was not favored under mechanochemical conditions.

The IR spectrum of the **1**–TEO powder com­plex and the IR spectrum of the single crystal were similar, indicating a structural homogeneity between the powder and the single crystal. The IR spectrum of **1** showed a single N—H band at 3283 cm^−1^. After the formation of the com­plex, the N—H band was red-shifted and split (suggesting asymmetry in the mol­ecule) into two bands with values of 3312 and 3293 cm^−1^ [Δν(N—H) = 10 and 29 cm^−1^, respectively]. The N—H band of TEO was also red-shifted as a consequence of the com­plex formation from 3120 to 3169 cm^−1^ [Δν(N—H) = 49 cm^−1^].

Concerning the carbonyl frequencies, com­pound **1** showed two bands at 1704 and 1688 cm^−1^, with Δν(C=O) = 12 and −4 cm^−1^. Theophylline showed Δν(C=O) = −5 and −24 cm^−1^.

**Figure 2:**
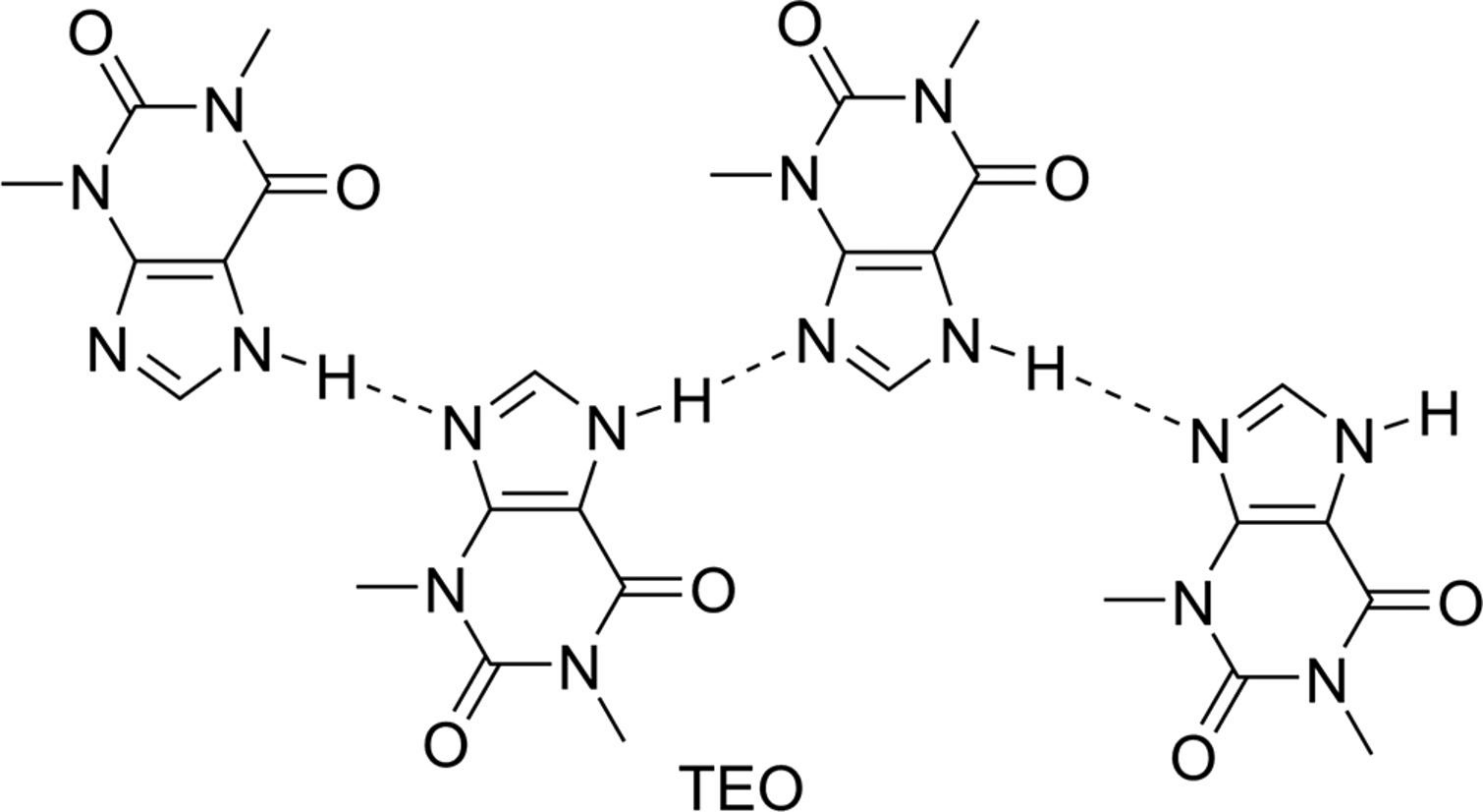
Hydrogen-bond patterns in free TEO.

The grinding process reorders the hydrogen-bonding patterns of the com­pounds involved in the formation of the com­plex shifting the C=O and N—H bands. Com­pound **1** is self-assembled by N—H⋯O=C hydrogen bonds [*C*(4) homosynthon] in the free form (see *Single-crystal X-ray diffraction*, §3.4). After the formation of the **1**–TEO com­plex, the N—H⋯O=C hydrogen-bond (heterosynthon) pattern is maintained; this explains the smaller values of Δν(N—H) and Δν(C=O) com­pared with the starting **1**. On the other hand, in the free form of TEO, the mol­ecules are inter­linked by N—H⋯N(imidazole) hydrogen bonds and π-inter­actions (Larkin *et al.*, 2014 ▸) (Fig. 2 ▸). The rearrangement of these hydrogen-bond patterns to form a new hydrogen-bond pattern results in greater Δν(N—H) and Δν(C=O) values of TEO with respect to the Δν(N—H) and Δν(C=O) values of **1**.

### Powder X-ray diffraction

**Figure 3:**
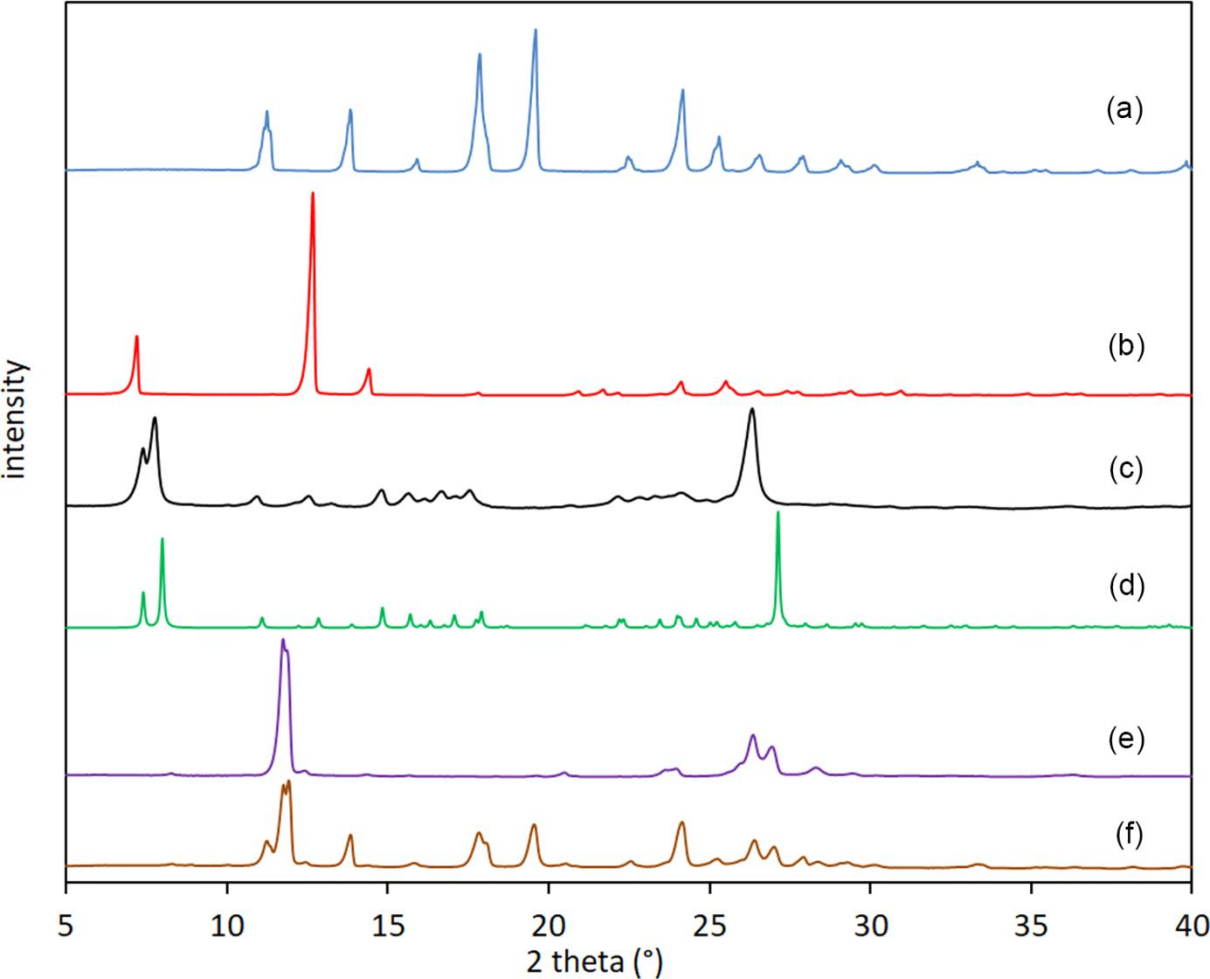
The powder X-ray diffractograms of (*a*) **1**, (*b*) TEO, (*c*) **1**–TEO ground powder, (*d*) the simulated pattern of **1**–TEO, (*e*) CAF and (*f*) **1**–CAF ground powder.

The powder X-ray diffraction patterns of the polycrystalline powder of **1**, solid TEO and CAF, and the polycrystalline powder of **1**–TEO and **1**–CAF were obtained. The solid form of TEO and CAF were identified as form II (Liu *et al.*, 2013 ▸; Mazel *et al.*, 2011 ▸) of each com­pound from the experimental powder diffraction pattern. The recorded powder pattern of **1** was similar to that simulated with *Mercury* (Macrae *et al.*, 2020 ▸) (Fig. S4), indicating structural homogeneity between the polycrystalline powder and the single crystal. The formation of the polycrystalline com­plex was evidenced because the PXRD diffraction pattern of the **1**–TEO polycrystalline ground powder was different com­pared with those of the starting materials (Fig. 3 ▸), showing new diffraction peaks at 2θ = 7.7, 14.8, 16.7 and 23.4°, and was similar to that simulated with *Mercury* (Macrae *et al.*, 2020 ▸). The absence of the signals at 2θ = 19.6 and 12.5° of starting **1** and TEO, respectively, in the powder pattern of **1**–TEO indicates the com­plete transformation of **1** and TEO to form the com­plex (Fig. 3 ▸). The PXRD pattern of **1**–CAF showed a combined pattern of **1** and CAF as a physical mixture [Fig. 3 ▸(*f*)] thus showing that the **1**–CAF com­plex was not formed.

### Solid-state ^13^C NMR

**Figure 4:**
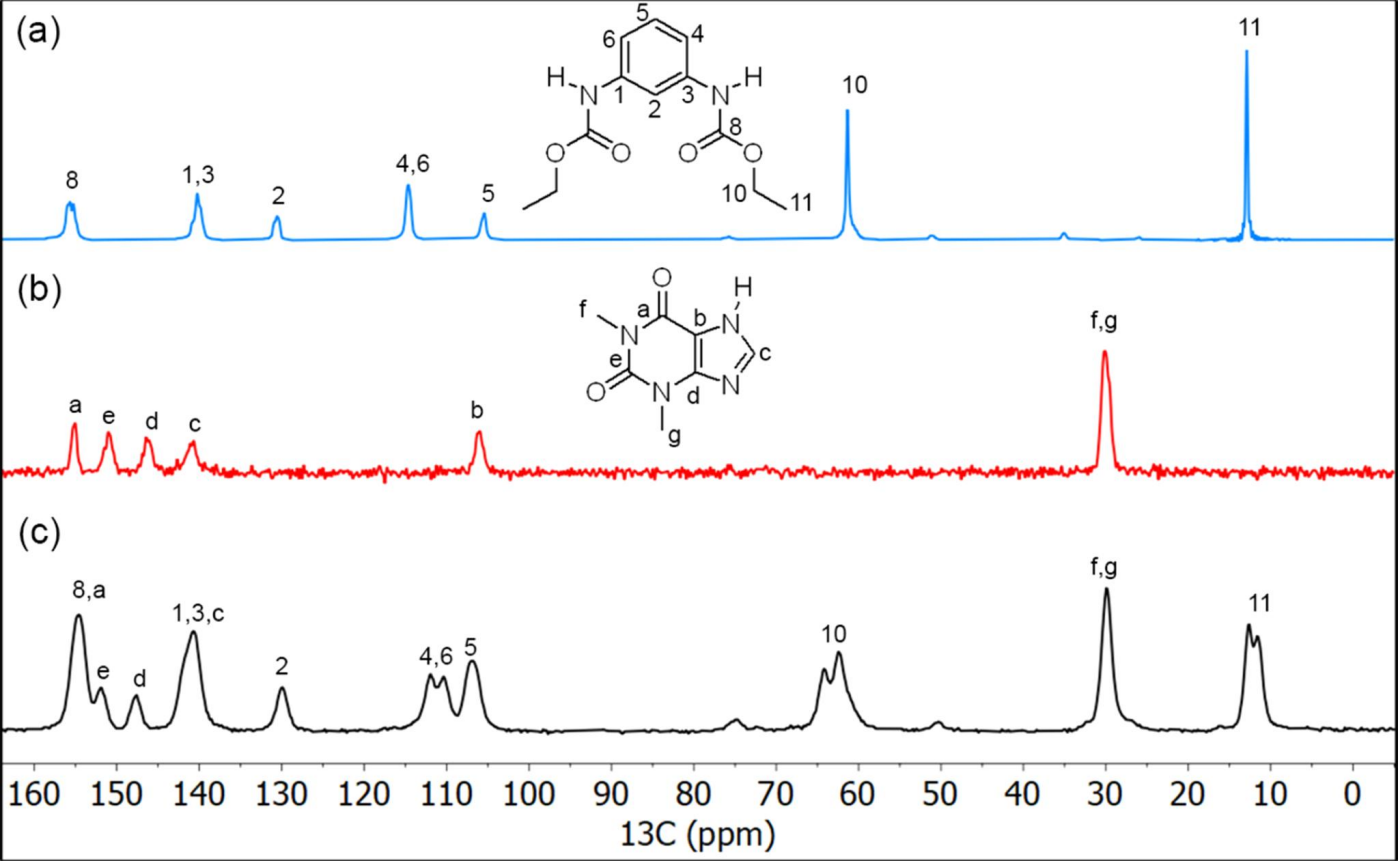
^13^C NMR spectra of (*a*) **1**, (*b*) TEO and (*c*) the **1**–TEO com­plex.

The solid-state ^13^C NMR spectra of **1**, TEO and the **1**–TEO powder com­plex were recorded (Fig. 4 ▸) and the ^13^C NMR assignments are listed in Table 3 ▸. Most of the signals in the ^13^C NMR spectrum of the **1**–TEO com­plex appeared shifted with respect to the starting com­pounds as a result of the change in the chemical environment due to the rearrangement of the hydrogen-bond patterns. The C=O signals were shifted from 155.9 to 154.5 ppm in **1** and from 150.9 to 151.9 ppm in TEO, indicating the formation of C=O⋯H—N hydrogen bonds between **1** and TEO. It worthy of mention that in the solid-state ^13^C NMR spectrum of **1**, only half of the signals were observed, indicating the presence of a *C*_2_ symmetry axis, which is consistent with the *endo–endo* conformation of **1**, as confirmed by single-crystal diffraction. Meanwhile, in the solid-state ^13^C NMR spectrum of the **1**–TEO com­plex, the signals of C10 and C11 from the ethyl group, and also the aromatic C4 and C6 signals, appeared split (Table 3 ▸), suggesting two crystallographically different ethyl groups originated from the adoption of the *exo–endo* conformation after the formation of the **1**–TEO com­plex.

### Single-crystal X-ray diffraction

**Figure 5:**
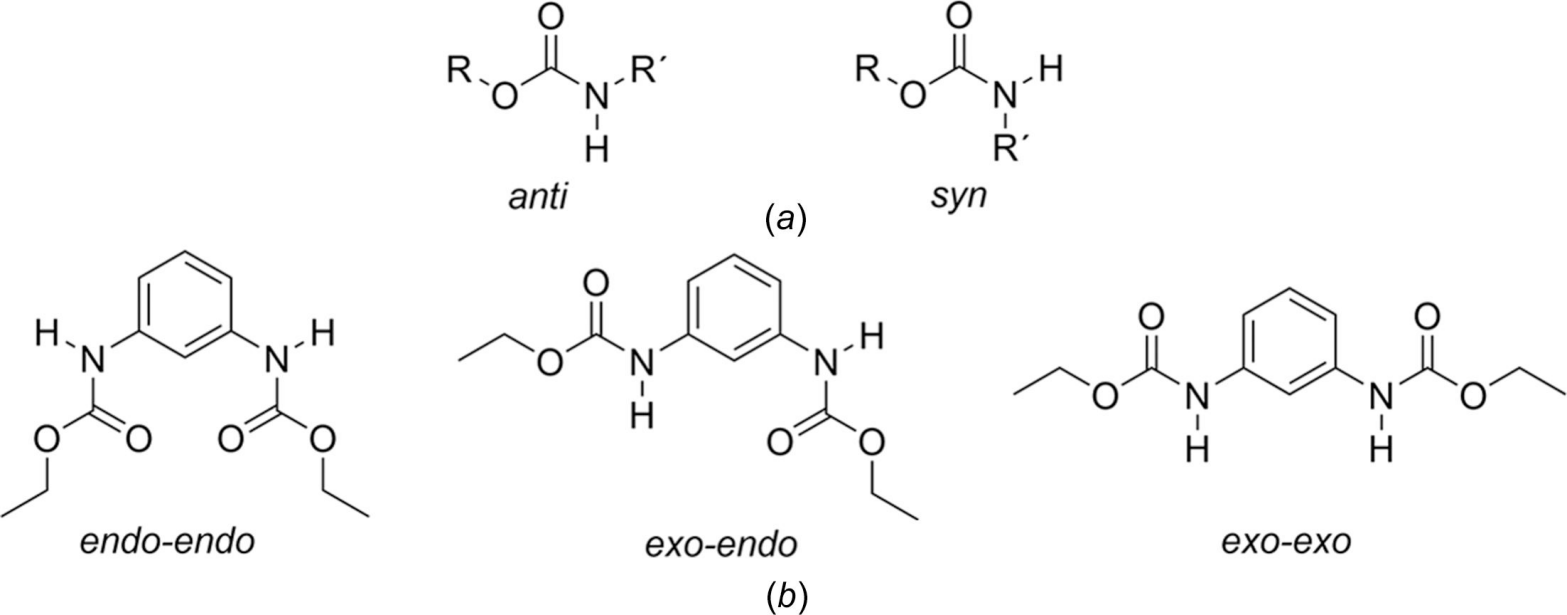
(*a*) Possible conformations of the carbamate group and (*b*) possible conformations of **1**.

The carbamate group in phenyl carbamates can adopt the *syn* or *anti* conformation according to the H7—N7—C8—O8 torsion angle [Fig. 5 ▸(*a*)]. A search of crystal structures in the Cambridge Structural Database (CSD, Version 5.45, update of November 2023; Groom *et al.* 2016 ▸) under the ‘phenyl­carbamate’ criteria, showed 98 results where the carbamate group adopts the *anti* conformation, and only one where the carbamate group adopts the *syn* conformation, *i.e.* the crystal structure of diisopropyl *N*,*N*′-(4-methyl-*m*-phenyl­ene)dicarbamate (CSD refcode JAYBUH; Lu *et al.*, 2005*b* ▸). Taking into consideration the cavity formed by the ethyl carbamate groups with respect to the benzene ring (torsion angle C6—C1—N7—C8), com­pound **1** can adopt the *endo–endo*, *exo–endo* and *exo–exo* conformations [Fig. 5 ▸(*b*)]. Four examples of crystal structures of 1,3-phenyl­enedicarbamates have been reported (Fig. S5): two adopt the *endo–endo* conformation [refcodes GAVGEQ (Lu *et al.*, 2005*a* ▸) and JAYBUH (Lu *et al.*, 2005*b* ▸)] and two adopt the *exo–exo* conformation [refcodes PIRQUG (Piper *et al.*, 2023 ▸) and OWOYIL (Alegre-Requena *et al.*, 2020 ▸)].

**Figure 6:**
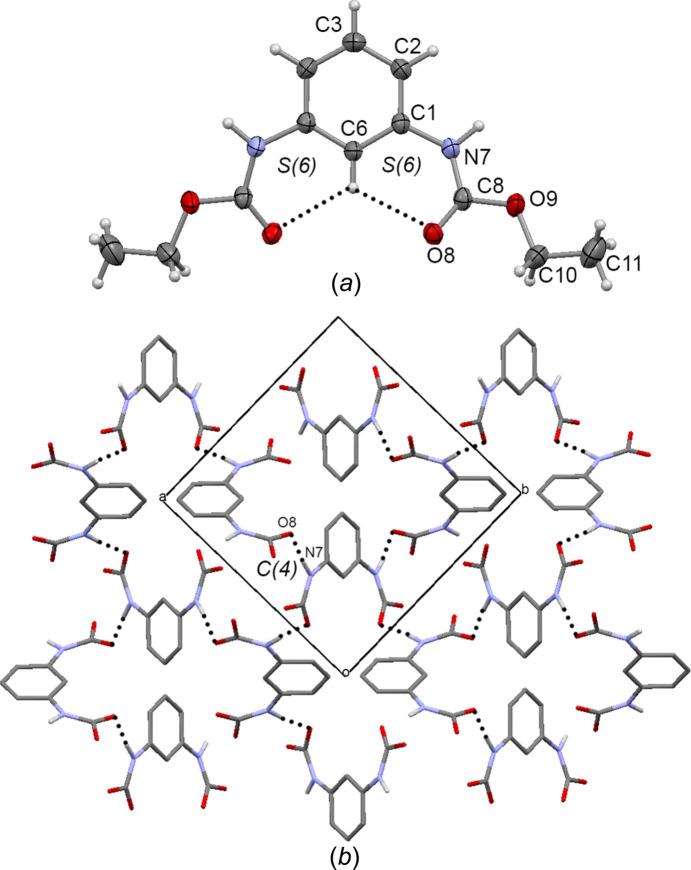
(*a*) The mol­ecular structure of **1**, with displacement ellipsoids drawn at the 30% probability level, showing the inter­molecular inter­actions. (*b*) The supra­molecular arrangement of **1** formed by N—H⋯O=C hydrogen bonds. Dashed lines represent hydrogen bonds. Some parts of the mol­ecules have been omitted for clarity. Dashed lines represent hydrogen bonds.

Com­pound **1** crystallized in the tetra­gonal space group *P*4_1_2_1_2, with the mol­ecule lying across a twofold axis having *C*_2_ symmetry; thus, only one half of the mol­ecule is present in the asymmetric unit. The crystal structure of **1** [Fig. 6 ▸ (*a*)] adopts the *endo–endo* conformation [with the C6—C1—N7—C8 torsion angle = −14.5 (4)°], reinforced by the formation of the C=O⋯H⋯O=C three-centred intra­molecular hydrogen bonds (C6—H6⋯O8 = 2.38 Å), depicting two adjacent *S*(6) motifs (the hydrogen-bond details and symmetry codes for **1** are given in Table 4 ▸). The ethyl carbamate group is twisted out from the plane of the benzene ring by 2.2 (4)° (C1—N7—C8—O8 torsion angle). The carbamate group adopts the *anti* conformation, with the H7—N7—C8—O8 torsion angle being 175.6 (2)°. Each mol­ecule of **1** is linked with four mol­ecules by N7—H7⋯O8 (1.97 Å) hydrogen bonding. This inter­action is extended along the *ab* plane to form a bidimensional supra­molecular arrangement depicting *C*(4) hydrogen-bond motifs [Fig. 6 ▸ (*b*)], as observed in GAVGEQ (Lu *et al.*, 2005*a* ▸), JAYBUH (Lu *et al.*, 2005*b* ▸) and PIRQUG (Piper *et al.*, 2023 ▸).

**Figure 7:**
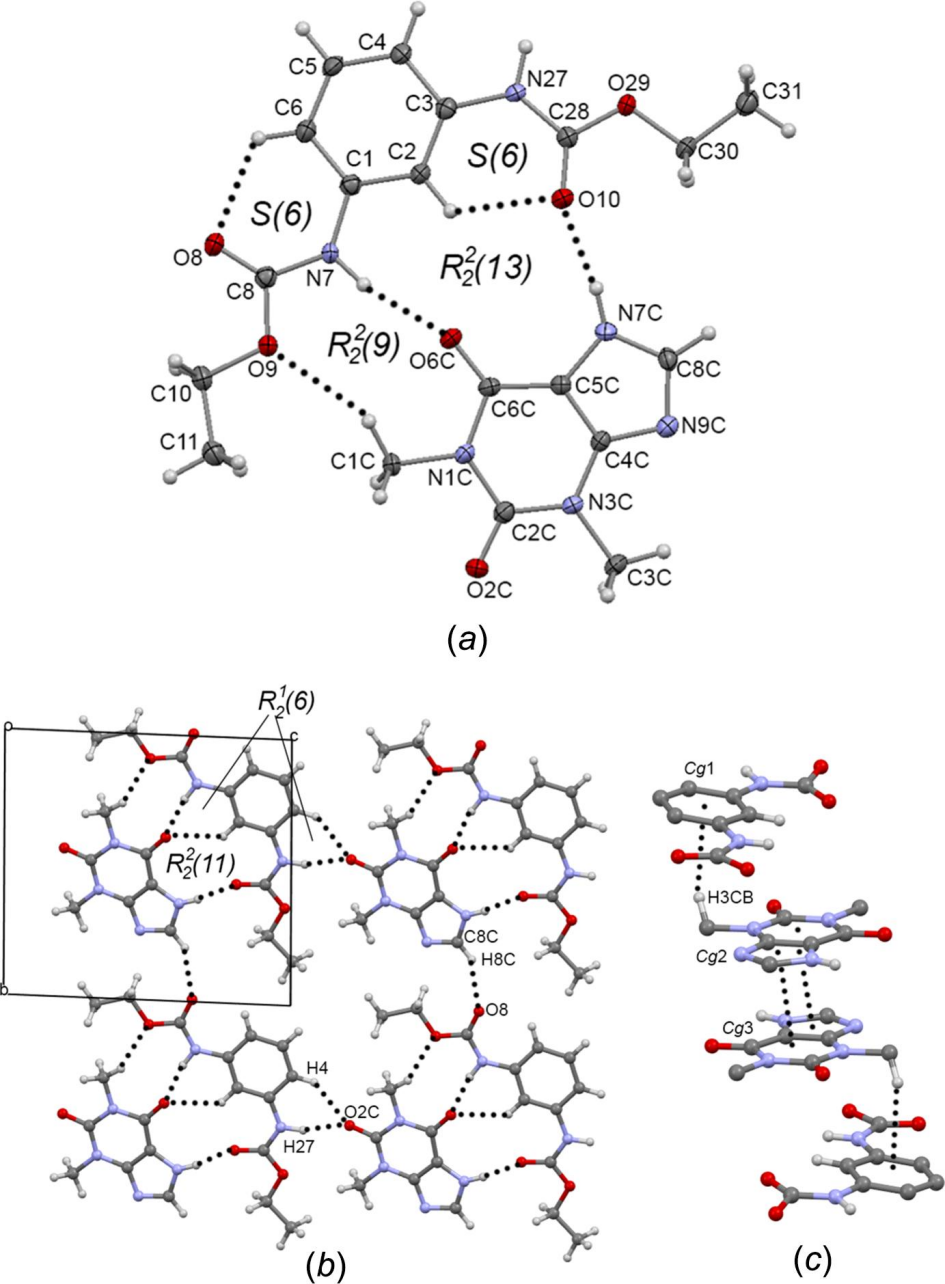
(*a*) The asymmetric unit, with displacement ellipsoids at the 30% probability level, of **1**–TEO, showing the atom numbering. (*b*) The supra­molecular sheet of **1**–TEO formed by the N27—H27⋯O2*C* ^iv^ and C8*C*—H8*C*⋯O8^v^ inter­actions. (*c*) π–π and C—H⋯π inter­actions found in **1**–TEO. Some parts of the mol­ecules have been omitted for clarity. Dashed lines represent hydrogen bonds or noncovalent inter­actions.

The **1**–TEO com­plex crystallized in the triclinic space group *P* 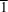 , the discrete unit consist of one mol­ecule of **1** and one mol­ecule of TEO [Fig. 7 ▸ (*a*)]. Receptor **1** adopts the *exo–endo* conformation, with torsion angles C2—C1—N7—C8 = 175.6 (2)° and C2—C3—N27—C28 = 2.8 (4)°, and the carbonyl group adopts the *syn* conformation, with torsion angles H7—N7—C8—O8 = 177.6 (2)° and H27—N27—C28—O10 = 177.9 (2)°.

**Figure 8:**
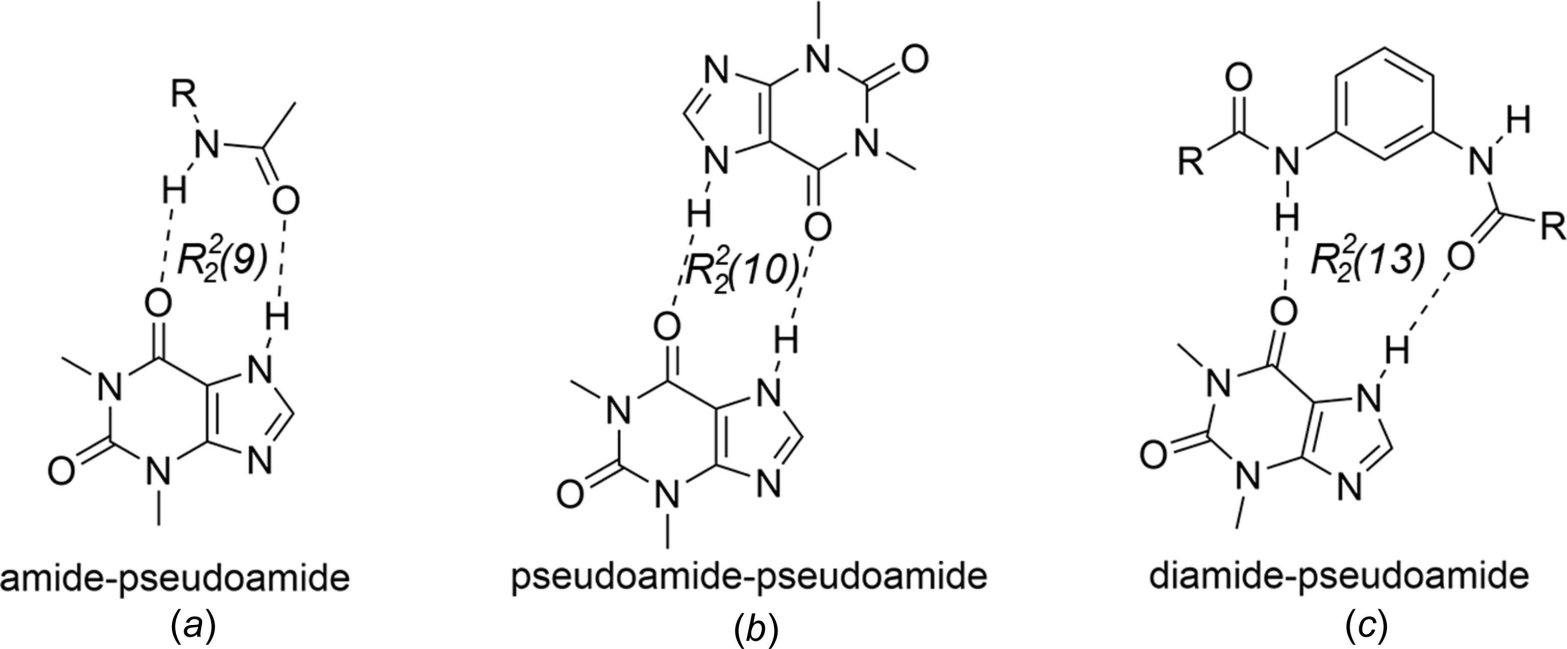
The observed synthons in TEO cocrystals.

The pseudo­amide fragment of the TEO mol­ecule (O6*C*—C6*C*—C5*C*—N7*C*—H7*C*) is involved in the formation of TEO cocrystals with amidic coformers (Eddleston *et al.*, 2016 ▸; Markad & Mandal, 2017 ▸). When the coformer is a primary or secondary amide group, the 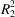 (9) amide-pseudo­amide synthon is formed [Fig. 8 ▸(*a*)], meanwhile the 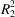 (10) pseudo­amide–pseudo­amide synthon consists of the self-assembly of two TEO mol­ecules [Fig. 8 ▸(*b*)], where the coformer is hydrogen bonded to TEO by the the urea carbonyl or the imidazole N atom. Receptor **1** and TEO are inter­linked by inter­molecular N—H⋯O=C hydrogen bonds [N7—H7⋯O6*C* = 2.02 (3) Å and N7*C*—H7*C*⋯O10 = 1.90 Å] depicting a new synthon, *i.e.* the 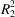 (13) ‘di­amide–pseudo­amide’ synthon [Fig. 8 ▸(*c*)] [this motif can be fragmented in two adjacent 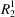 (6) and 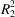 (11) motifs, including the C2—H2⋯O6*C* inter­action] [Fig. 7 ▸(*b*)]. The com­plementary C1*C*—H1*CB*⋯O9 (2.49 Å) inter­action, depicting an 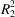 (11) motif, is also involved in the inter­connection of **1** and TEO. The angle between the planes formed by the benzene ring and the TEO mol­ecule is 9.42°, indicating that **1** and TEO are almost coplanar and the good fit of TEO into the cavity formed by the ethyl carbamate groups. The intra­molecular C2—H2⋯O10 *S*(6) inter­action becomes shorter (2.22 Å) com­pared with starting **1** (2.38 Å). The observed inter­molecular inter­actions between **1**–TEO units, *i.e.* the N27—H27⋯O2*C* = 1.96 (2) Å hydrogen bond, and the C4—H4⋯O2*C* = 2.44 Å [ 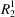 (6) motif] and C8—H8*C*⋯O8*C* = 2.48 Å inter­actions, give rise to a bidimensional supra­molecular sheet extended along the *bc* plane [Fig. 7 ▸(*b*)]. Supra­molecular sheets are connected by π-stacking of TEO (*Cg*2⋯*Cg*3 = 3.35 Å; *Cg*2 and *Cg*3 are the centroids of the N7*C*/C5*C*/C4*C*/N9*C*/C8*C* and N1*C*/C2*C*/N3*C*/C4*C*/C5*C*/C6*C* rings, respectively) and C—H⋯π inter­actions (C3—H3*CB*⋯*Cg*1 = 2.87 Å; *Cg*1 is the centroid of the C1/C2/C3/C4/C5/C6 ring) [Fig. 7 ▸(*c*)].

### Conformational change of 1 and selective binding of TEO

The mol­ecular structure of starting **1** adopts the *endo–endo* conformation, showing a single N—H band in the IR spectrum and half of the signals in the solid-state ^13^C NMR spectrum. The formation of the **1**–TEO com­plex by mechanochemical grinding involves the conformational change of **1** from the *endo–endo* conformation to the *exo–endo* conformation (showing two N—H bands in the IR spectrum and the split of the ethyl signals in the solid-state ^13^C NMR spectrum of **1**–TEO), while the grinding of **1** and CAF under the same conditions used to obtain **1**–TEO did not result in the formation of the **1**–CAF com­plex.

**Figure 9:**
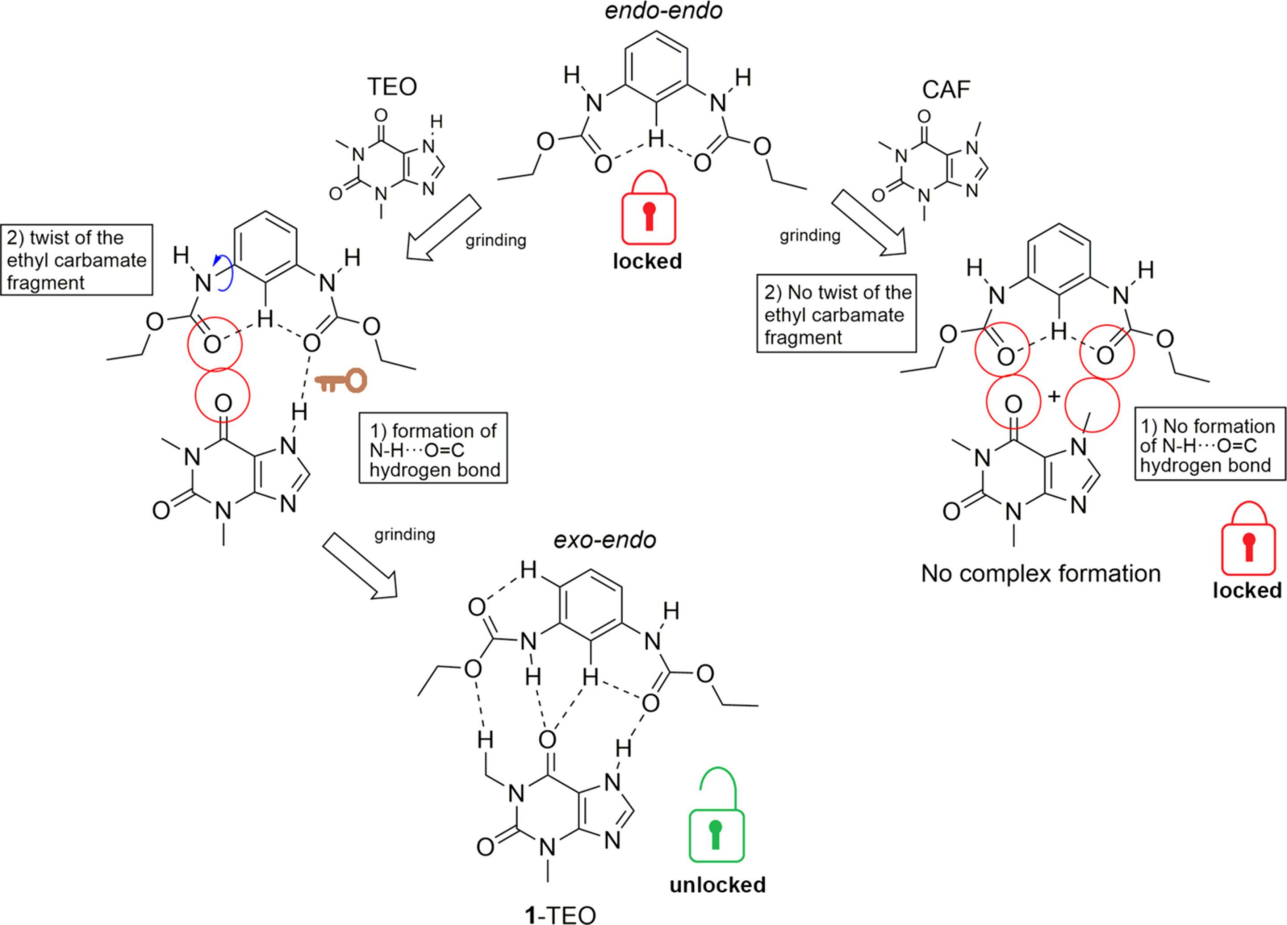
Conformational change of **1** in the formation of the **1**–TEO com­plex and the lack of conformational change of **1** in the **1**–CAF ground mixture.

In the *endo–endo* conformation, a potential carbon­yl–carbonyl repulsive effect avoids the com­plex formation by adopting a ‘locked’ state (Fig. 9 ▸). The formation of the **1**–TEO com­plex implies that grinding provides the energy necessary for the rotation of one of the ethyl carbamate groups to adopt the *exo–endo* conformation of the ‘unlocked’ state (Fig. 9 ▸) [conformational change after com­plexation from the *exo* to the *endo* conformation (González-González *et al.*, 2014 ▸), and from the *endo* to the *exo* conformation (González-González *et al.*, 2015 ▸) is also observed in the formation of mol­ecular com­plexes of diethyl *N*,*N*′-1,3-phenyl­enedioxalamates with 1,3-benzene­diols], allowing the formation of inter­molecular hydrogen bonds between **1** and TEO. On the other hand, the IR spectrum and the PXRD pattern of the **1**–CAF ground mixture indicated that the **1**–CAF com­plex was not formed and receptor **1** remains in the ‘locked’ state (*endo–endo* conformation).

**Figure 10:**
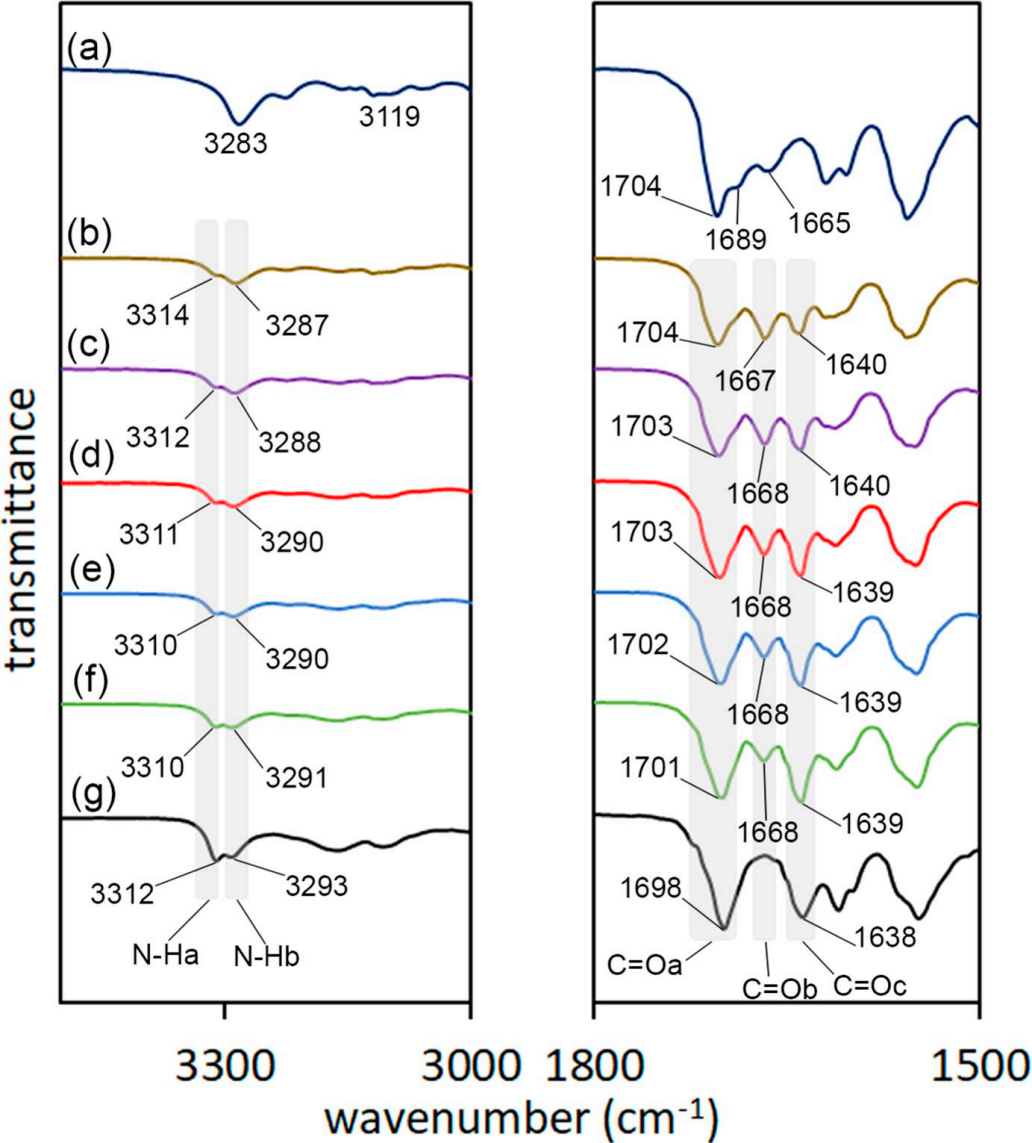
Partial IR spectra of (*a*) the physical mixture of **1** and TEO. Polycrystalline powder of **1**–TEO after (*b*) 3 min of grinding, (*c*) 6 min of grinding, (*d*) 9 min of grinding, (*e*) 12 min of grinding and (*f*) 15 min of grinding. (*g*) The IR spectrum of the single crystal of **1**–TEO.

To obtain information about the possible mechanism of the conformational change of **1** to form the **1**–TEO com­plex and the preference of receptor **1** to link TEO over CAF, firstly, the mechanochemical grinding of **1** (in the *endo–endo* form), under the same conditions to obtain the **1**–TEO com­plex (12 min of grinding time adding di­chloro­methane), was performed. The IR spectrum of **1** after 12 min of grinding time remained unchanged (Fig. S6 in the supporting information), indicating that the mechanochemical energy of the grinding is not able to drive the conformational change of free **1**. A second strategy was to perform the mechanochemical grinding of **1** and TEO without solvent to retard the formation of the com­plex, and com­pare the IR spectra of the obtained ground powder with the IR spectrum of the physical mixture and with the IR spectrum of the **1**–TEO single crystal (Fig. 10 ▸). The IR spectrum of the physical mixture showed the N—H bands at 3283 cm^−1^ for **1** and at 3119 cm^−1^ for TEO; meanwhile, the C=O bands were observed at 1704 and 1688 cm^−1^ for **1**, and at 1665 for TEO. After 3 min of ‘dry’ grinding, the obtained IR spectrum showed two N—H bands: a shoulder band at 3314 cm^−1^ (N—Ha) and the principal N—H band of **1** at 3287 cm^−1^ (N—Hb). The presence of two N—H bands of **1** (as in the IR spectrum of the **1**–TEO single crystal) indicates the asymmetry of the mol­ecule by the conformational change of one of the ethyl carbamate fragments, adopting the *exo–endo* conformation. The carbonyl region showed three bands: (i) a band at 1704 cm^−1^ (C=Oa) belonging to **1**; (ii) a band at 1667 cm^−1^ (C=Ob) for TEO; and (iii) a band at 1640 cm^−1^ (C=Oc) which is present in the **1**–TEO com­plex. Here, the C=Ob band is slightly more intense than C=Oc, indicating that after 3 min of grinding, part of TEO remains free, and the com­plex has started to be formed. The IR spectra obtained after 6, 9, 12 and 15 min of ‘dry’ grinding showed the following: the intensity of the N—Ha band increased as a signal of the formation of the com­plex and the N—Hb band was red shifted; the intensity of the C=Oa band remained unchanged. As the **1**–TEO com­plex was formed, the intensity of the C=Ob band of TEO at 1668 cm^−1^ decreased; meanwhile, the intensity of the C=Oc band increased. This indicates that the presence of TEO and the mechanochemical grinding induces the rotation of the ethyl carbamate group of **1** and ‘unlocks’ the *endo–endo* conformation to allow the formation of inter­molecular inter­actions between **1** and TEO to form the com­plex (Fig. 9 ▸). The formation of the (TEO)N—H⋯O=C(**1**) hydrogen-bond inter­action acts as the ‘key’ that unlocks the *endo–endo* conformation and then the ethyl carbamate group rotates (to the ‘unlocked’ state) to allow the formation of the rest of the inter­molecular inter­actions and form the di­amide–pseudo­amide 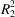 (13) synthon in the *exo–endo* conformation (Fig. 9 ▸). On the other hand, CAF is unable to form the N—H⋯O=C ‘key’ hydrogen bond because it possesses an N—CH_3_ group instead of the N—H group in TEO, avoiding the formation of the **1**–CAF com­plex in the same way as **1**–TEO (almost coplanar with respect to the plane of the benzene ring). It is important to mention that in the urea–CAF cocrystal and the host–guest com­plexes of CAF with tri­phenyl­ene ketal triurea-based receptors, CAF acts as a hydrogen-bond acceptor, forming N—H⋯O⋯H—N and N—H⋯N⋯H—N hydrogen bonds where the urea group is positioned perpendicular with respect to the plane of the CAF mol­ecule, unlike the **1**–TEO com­plex where **1** and TEO are coplanar (MacFhionnghaile *et al.*, 2020 ▸; Fiammengo *et al.*, 2003 ▸; Schopohl *et al.*, 2005 ▸).

## Conclusions

The ability of receptor **1** to form host–guest com­plexes with TEO and CAF by mechanochemistry was evaluated, resulting only in the formation of the **1**–TEO com­plex involving a conformational change of **1**, in which one of the ethyl carbamate groups changes from the *endo* conformation to the *exo* conformation to allow the formation of noncovalent inter­actions between **1** and TEO. An IR spectroscopy study revealed that the (TEO)N—H⋯O=C(**1**) hydrogen bond triggers the rotation of the ethyl carbamate group from the *endo* conformation to the *exo* conformation. The formation of the **1**–CAF com­plex was not possible because CAF possesses an N—CH_3_ group instead of the N—H group in TEO, thus avoiding the formation of the N—H⋯O=C hydrogen bond. The formation of **1**–TEO was evidenced by the shift of the N—H and C=O frequencies in the **1**–TEO powder com­plex, and by the shifts in the solid-state ^13^C NMR signals com­pared with the IR and ^13^C NMR spectra of the starting materials, suggesting the formation of N—H⋯O=C hydrogen bonds. The formation of the new polycrystalline phase was confirmed because the powder X-ray diffraction pattern of **1**–TEO was different from those of the starting **1** and TEO. Single-crystal X-ray diffraction analysis showed that **1** adopts the *endo–endo* conformation in the solid state and is self-assembled by N—H⋯O=C hydrogen bonds; meanwhile, the mol­ecular structure of the **1**–TEO com­plex showed a 1:1 stoichiometric ratio, where **1** and TEO are inter­linked by N—H⋯O=C hydrogen bonds and C—H⋯O inter­actions, and **1** adopts the *exo–endo* conformation, exhibiting the di­amide–pseudomide 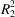 (13) synthon. The supra­molecular architecture of **1**–TEO is driven by N—H⋯O=C hydrogen bonds and π–π and C—H⋯π inter­actions.

## Supplementary Material

Crystal structure: contains datablock(s) 1, 1_TEO, global. DOI: 10.1107/S2053229624003358/zo3045sup1.cif

Structure factors: contains datablock(s) 1. DOI: 10.1107/S2053229624003358/zo30451sup2.hkl

Structure factors: contains datablock(s) 1_TEO. DOI: 10.1107/S2053229624003358/zo30451_TEOsup3.hkl

Supporting information file. DOI: 10.1107/S2053229624003358/zo30451sup4.cml

Supporting information file. DOI: 10.1107/S2053229624003358/zo30451_TEOsup5.cml

Supporting information file. DOI: 10.1107/S2053229624003358/zo3045sup6.pdf

CCDC references: 2328711, 2328710

## Figures and Tables

**Table 1:** Experimental details Experiments were carried out with Mo *K*α radiation using a Agilent Xcalibur Atlas Gemini diffractometer. The absorption correction was analytical (*CrysAlis PRO*; Agilent, 2013 ▸). H atoms were treated by a mixture of independent and constrained refinement.

	**1**	**1**–TEO
Crystal data		
Chemical formula	C_12_H_16_N_2_O_4_	C_12_H_16_N_2_O_4_·C_7_H_8_N_4_O_2_
*M*_r_	252.27	432.44
Crystal system, space group	Tetragonal, *P*4_1_2_1_2	Triclinic, *P* 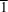
Temperature (K)	298	130
*a*, *b*, *c* (Å)	11.1312 (13), 11.1312 (13), 10.894 (3)	7.5284 (14), 11.2362 (18), 12.2606 (12)
α, β, γ (°)	90, 90, 90	85.742 (10), 76.887 (12), 79.803 (15)
*V* (Å^3^)	1349.8 (5)	993.5 (3)
*Z*	4	2
μ (mm^−1^)	0.09	0.11
Crystal size (mm)	0.41 × 0.33 × 0.3	0.38 × 0.12 × 0.07

Data collection		
*T*_min_, *T*_max_	0.972, 0.976	0.976, 0.993
No. of measured, independent and observed [*I* > 2σ(*I*)] reflections	4765, 1620, 1153	11140, 4780, 3041
*R*_int_	0.027	0.051
(sin θ/λ)_max_ (Å^−1^)	0.693	0.697

Refinement		
*R*[*F* ^2^ > 2σ(*F* ^2^)], *wR*(*F* ^2^), *S*	0.047, 0.128, 1.05	0.062, 0.157, 1.05
No. of reflections	1620	4780
No. of parameters	87	290
Δρ_max_, Δρ_min_ (e Å^−3^)	0.12, −0.14	0.29, −0.38
Absolute structure	Flack *x* determined using 343 quotients [(*I* ^+^) − (*I* ^−^)]/[(*I* ^+^) + (*I* ^−^)] (Parsons *et al.*, 2013 ▸)	–
Absolute structure parameter	−1.9 (9)	–

Com­puter programs: *CrysAlis PRO* (Agilent, 2013 ▸), *SHELXT2018* (Sheldrick, 2015*a* ▸), *SHELXL2018* (Sheldrick, 2015*b* ▸), *ORTEP-3 for Windows* (Farrugia, 2012 ▸) and *WinGX* (Farrugia, 2012 ▸).

**Table 2:** IR frequencies (cm^−1^)

Com­pound	N—H	ΔN—H	C=O	ΔC=O
**1**	3283	–	1704, 1688	–
TEO	3120	–	1705, 1662	–
**1**–TEO_ground_	3312, 3293, (3169)	29, 10, (49)	1700, (1638)	(−5), 12, −4*, (−24)
**1**–TEO_cryst_	3309, 3292, (3162)	26, 9, (42)	1698, (1638)	(−7), 10, −6, (−24)
CAF	–	–	1694, 1645	–
**1**–CAF_ground_	3284	1*	1705, 1692, (1658)	1*, 4*, (13)**

Notes: (*) under spectral resolution; (**) the apparent shift of the band is due the change of the curvedness of the C=O band in the IR spectra. Values in brackets belong to the IR frequencies of TEO or CAF.

**Table 3:** Solid-state ^13^C chemical shifts of **1**, TEO and **1**–TEO (δ = ppm)

	C1,C3	C2	C4,C6	C8	C9	C10	C*a*	C*b*	C*c*	C*d*	C*e*	C*f,g*
**1**	140.1	130.4	114.6	155.9	61.2	12.8	–	–	–	–	–	–
TEO	–	–	–	–	–	–	154.9	105.8	140.5	146.3	150.9	30.0
**1**–TEO	140.7	129.9	111.9, 110.3	154.5	64.1, 62.4	12.6, 11.6	154.5	106.8	140.7	147.6	151.9	29.9

**Table 4:** Hydrogen-bond geometry (Å, °) for **1** and **1**–TEO

	*D*—H⋯*A*	*D*—H	H⋯*A*	*D*⋯*A*	*D*—H⋯*A*
**1**	N7—H7⋯O8^i^	0.93 (3)	1.97 (3)	2.897 (3)	176 (3)
	C6—H6⋯O8	0.93	2.38	2.950 (3)	119
	C6—H6⋯O8^ii^	0.93	2.38	2.950 (3)	119
**1**–TEO	N7—H7⋯O6^iii^	0.91 (3)	2.02 (3)	2.920 (3)	168 (2)
	N7—H7*C*⋯O10^iii^	0.88	1.90	2.770 (3)	172
	N27—H27⋯O2*C* ^iv^	0.94 (2)	1.96 (2)	2.877 (2)	167 (2)
	C2—H2*C*⋯O6*C* ^iii^	0.95	2.52	3.306 (3)	140
	C1*C*—H1*CB*⋯O9^iii^	0.98	2.49	3.399 (3)	153
	C4—H4C⋯O2*C* ^iv^	0.95	2.44	3.219 (3)	139
	C8—H8*C*⋯O8^v^	0.95	2.48	3.303 (3)	145
	C1*C*—H1*CC*⋯O10^vi^	0.98	2.57	3.400 (3)	142
	C2—H2⋯O10	0.95	2.23	2.849 (3)	122
	C6—H6⋯O8	0.95	2.29	2.895 (3)	121

Symmetry codes: (i) *y* + 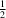 , −*x* + 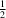 , *z* − 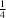 ; (ii) *y*, *x*, −*z* + 1: (iii) −*x* + 1, −*y* + 1, −*z* + 1; (iv) −*x* + 1, −*y* + 1, −*z*; (v) −*x*, −*y* + 2, −*z* + 1; (vi) *x*, *y*, *z*.
