# Supplementary material for: Mol­ecular structure and selective theophylline com­plexation by conformational change of diethyl N,N′-(1,3-phenyl­ene)dicarbamate
Source: Acta Crystallogr C Struct Chem. 2024 May 7;80(Pt 6):190–9. doi: 10.1107/S2053229624003358 (PMC11150875; doi:10.1107/S2053229624003358)
Supplement: Supplementary file 6 [file c-80-00190-sup6.pdf]

STRUCTURAL CHEMISTRY

Volume 80 (2024)

Supporting information for article:

Molecular structure and selective theophylline complexation by conformational change of diethyl *N*,*N*′-(1,3-phenylene)dicarbamate

Juan Saulo González-González, Alfonso Martínez-Santos, María José Emparán-Legaspi, Armando Pineda-Contreras, Francisco Javier Martínez-Martínez, Marcos Flores-Alamo and Hector García-Ortega

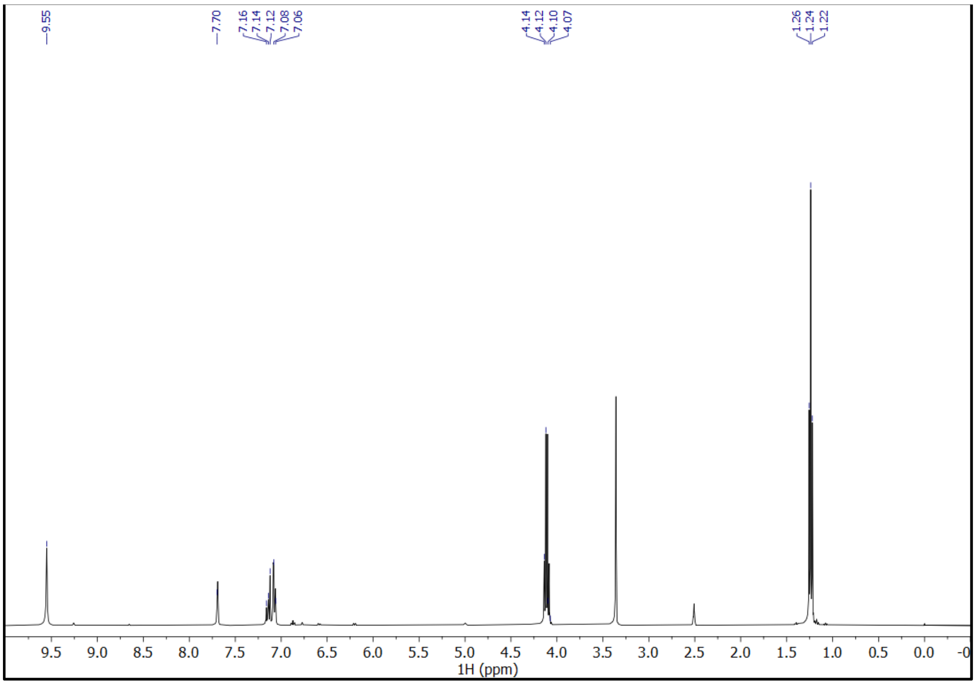

Figure S1 ^{1}H NMR spectrum of **1** in DMSO d_6 (400 MHz).

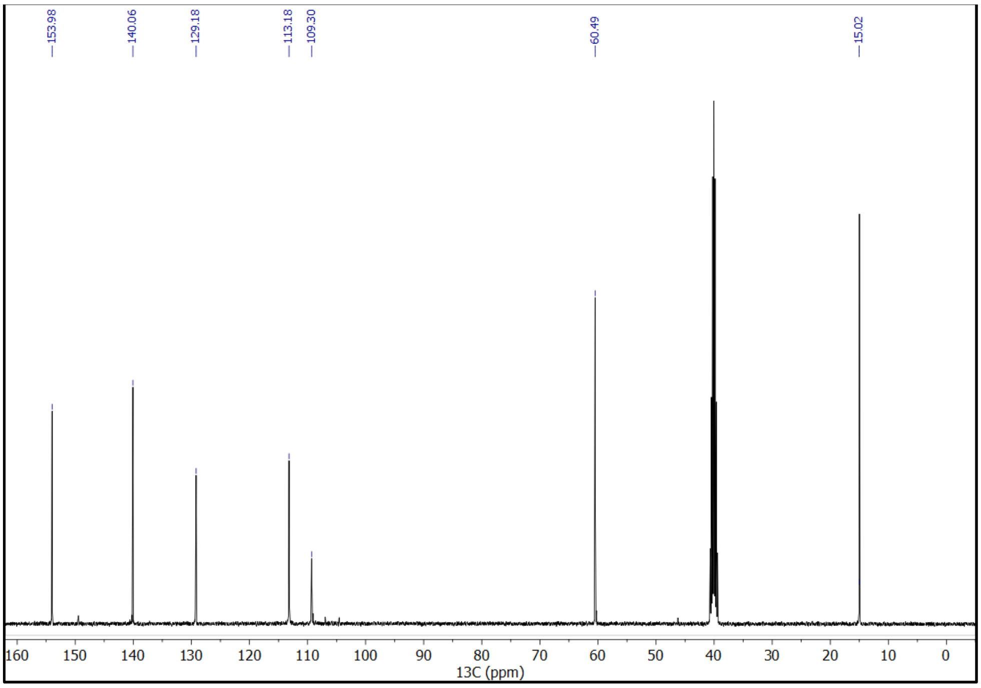

Figure S2 ^{13}C NMR spectrum of **1** in DMSO d_6 (100 MHz).

Document: 12-09-2014 (VarioMICRO) from: 12/09/2014 18:19:23

Centro Conjunto de Investigación en Química Sustentable
Muestra: UNCA-03 Realizó: Alejandra Núñez
Teóricos: 11.10%N 57.13%C 6.39%H No.reg. 1611

Text report

No.	Name	Weight [mg]	N [%]	C [%]	H [%]
38	UNCA-03	1.5340	11.04	56.82	6.39

Figure S3 Elemental analysis report of **1.**

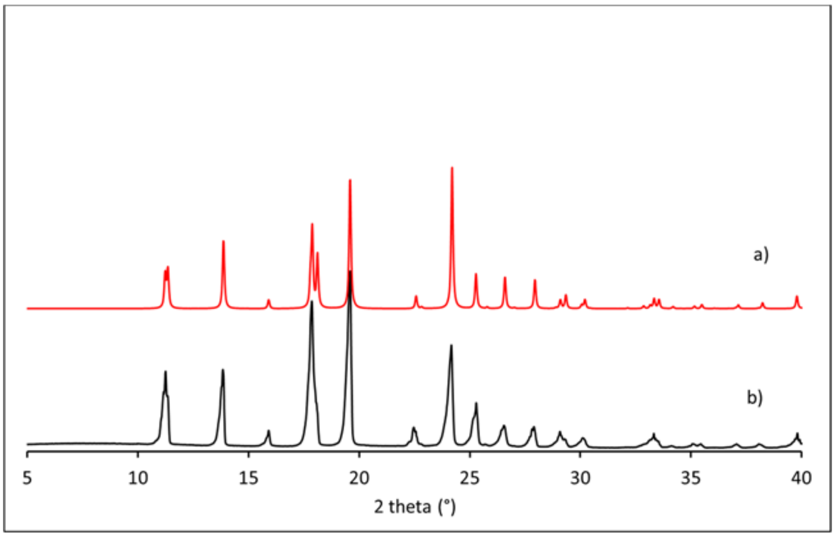

Figure S4 a) powder X-ray simulated pattern of **1**; b) experimental powder X-ray pattern of **1**.

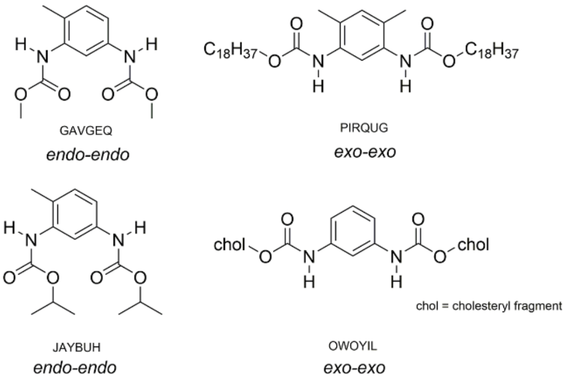

Figure S5 1,3-phenylene dicarbamates reported in the CSD.

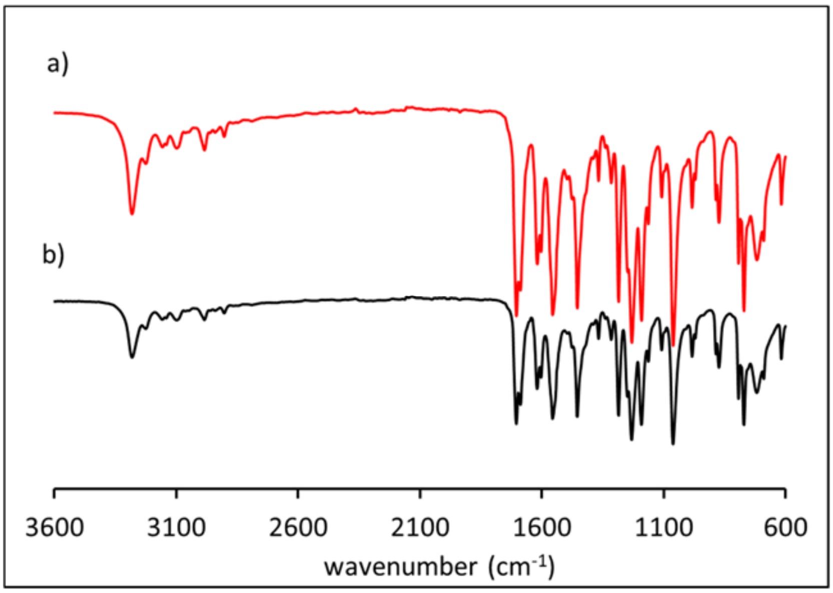

Figure S6 a) IR spectrum of **1**; b) IR spectrum **1** after 12 min. of grinding.
